# Decidual natural killer cells regulate vessel stability: implications for impaired spiral artery remodelling

**DOI:** 10.1016/j.jri.2015.04.003

**Published:** 2015-08

**Authors:** Rupsha Fraser, Guy St.J. Whitley, Baskaran Thilaganathan, Judith E. Cartwright

**Affiliations:** a Institute of Cardiovascular and Cell Sciences, St. George's, University of London, Cranmer Terrace, London SW17 0RE, United Kingdom; b Fetal Medicine Unit, St. George's Hospital, Blackshaw Road, London SW17 0QT, United Kingdom

**Keywords:** Decidua, Natural killer, Endothelium, Spiral artery, Pre-eclampsia, Remodelling

## Abstract

Decidual NK (dNK) cells are present during uterine spiral artery remodelling, an event that is crucial for successful placentation and the provision of an adequate blood supply to the developing fetus. Spiral artery remodelling is impaired in the pregnancy complication pre-eclampsia. Although dNK cells are known to play active roles at the maternal–fetal interface, little is known about their effect on endothelial integrity, an important component of vessel stability. We present a study in which we have modelled dNK–endothelium interactions, using first-trimester dNK cells isolated from both normal pregnancies and those with impaired spiral artery remodelling. dNK cells were isolated from first-trimester pregnancies, screened by uterine artery Doppler ultrasound to determine resistance indices (RI) that relate to the extent of spiral artery remodelling. dNK culture supernatant from normal-RI pregnancies (but not high-RI pregnancies) destabilised endothelial tube-like structures in Matrigel, and normal-RI dNK cells induced endothelial intercellular adhesion molecule-1 and tumour necrosis factor-α expression to a greater extent than high-RI dNK cells. We have established a functional role for dNK cells in the disruption of endothelial structures and have suggested how impairment of this process may be contributing to the reduced vessel remodelling in pregnancies with a high uterine artery resistance index. These findings have implications for our understanding of the pathology of pre-eclampsia and other pregnancy disorders where remodelling is impaired.

## Highlights

• dNK cells isolated from pregnancies with high or normal uterine artery resistance indices.
• Normal-RI dNK cells activate ECs and destabilise endothelial tube-like structures.
• Normal-RI dNK cells induce more TNFα production from ECs than high-RI dNK cells.
• TNFα destabilises endothelial tube-like structures.

## 1 Introduction

The human uterus undergoes extensive vascular remodelling. Before implantation, angiogenic events occur in the endometrium, as part of the decidualisation process. Following implantation, specialised fetally derived cells of the placenta, the extravillous trophoblast (EVT), invade the decidua and remodel the maternal uterine arteries by removing and replacing the vascular cells that line the arteries (Pijnenborg et al., 2006). During early pregnancy, the uterine spiral arteries are remodelled into larger diameter, higher flow vessels, allowing a 10-fold increase in blood supply into the intervillous space for placental uptake. This is critical for the developing fetus to obtain sufficient oxygen and nutrients; and incomplete spiral artery remodelling can result in the dangerous hypertensive pregnancy disorder, pre-eclampsia (PE) (Brosens et al., 1972).

The mechanisms responsible for the remodelling events in normal pregnancy are beginning to be elucidated, suggesting that spiral artery remodelling might not only be reliant on the EVT, but might also be regulated by the large infiltration of maternal immune cells present in the decidua. Decidual natural killer (dNK) cells comprise approximately 70% of the decidual leukocyte population and, unlike peripheral blood NK cells, are a cytokine-producing cell type with limited cytotoxic capacity. dNK cells accumulate around spiral arteries, are present ahead of trophoblast cell invasion and continue to be present during the remodelling process (Smith et al., 2009). It has been suggested that dNK cells might be involved in angiogenesis during decidualisation (Hanna et al., 2006; Blois et al., 2011), regulation of trophoblast invasion (Wallace et al., 2013) and spiral artery remodelling (Fraser et al., 2012).

The evidence to date suggests that dNK cells might play an active role in the establishment of appropriately transformed spiral artery structures at the maternal–fetal interface in human pregnancy. Histological studies performed on human decidua have identified both trophoblast-dependent and independent stages of remodelling (Craven et al., 1998). The first stages of vascular remodelling are apparent in the spiral artery endothelium, with signs of endothelial cell (EC) activation and vacuolisation, and in the vascular smooth muscle layer, where there is disorganisation and the start of fibrinoid deposition. Some of these changes occur before vascular cell contact with the invading EVT (Craven et al., 1998) and occur in the presence of leukocytes, but not trophoblasts (Craven et al., 1998; Smith et al., 2009; Hazan et al., 2010). The latter stages of remodelling, where there is removal of vascular smooth muscle cells (VSMCs) and temporary replacement of ECs with trophoblast, are likely to involve both trophoblast-dependent and immune cell-dependent changes (Harris et al., 2006; Keogh et al., 2007; Harris, 2010; Hazan et al., 2010; Fraser et al., 2012). As dNK cells are the most abundant decidual leukocyte population, it is likely that these cells influence vascular structure both in the early stages of remodelling and then co-operate with invading EVT in the latter stages (Wallace et al., 2012, 2013, 2014).

The alterations in vessel wall architecture that occur during vascular remodelling are likely to be regulated by interactions between the cell types that form the vessel structure itself, as well as those present in the vicinity of the vessel (Bennett et al., 2012). ECs are able to sense stimuli that induce remodelling, both from the lumen of the vessel (such as haemodynamic stress) and within the vessel wall (such as cytokine signalling from VSMCs), or via immune cells located in their microenvironment. Although it is known that the endothelium changes considerably in its activation and stability, the regulation of this process by decidual leukocytes has not been investigated.

We have used dNK cells isolated from women undergoing elective termination of pregnancy at 9–14 weeks’ gestation to investigate their role in both the establishment of a healthy pregnancy and the pathogenesis of complications where remodelling is impaired. These pregnancies have been classified by Doppler ultrasound scanning of the uterine arteries, a proxy measure of the extent of spiral artery remodelling/successful placentation. We have modelled dNK–endothelium interactions at the maternal–fetal interface, using dNK cells isolated from both normal and aberrantly remodelled early human pregnancies.

## 2 Materials and methods

### 2.1 Doppler ultrasound of uterine artery resistance

Maternal uterine artery Doppler ultrasound scans were conducted on women attending clinic for elective termination of pregnancy at 9–14 weeks of gestation as previously described (Melchiorre et al., 2008). Wandsworth Local Research Ethics Committee approval was in place for both the Doppler ultrasound and donation of tissue after surgical termination (ethical committee references: 01.96.8 and 01.78.5), and all women gave informed written consent. Gestational age was calculated by crown–rump length measurement. All were singleton pregnancies, with no pre-existing medical conditions. High resistance index (high-RI) cases were defined as those presenting with bilateral uterine diastolic notches and a mean RI above the 95th percentile. Normal-RI cases were defined as presenting with no diastolic notches and a mean RI below the 95th percentile. Abnormal uterine artery Doppler in the first trimester is associated with deficient trophoblast invasion of spiral arteries (Prefumo et al., 2004). The normal-RI cases represent the least (<1%), while the high-RI cases represent the most (21%) likely to have developed pre-eclampsia, had the pregnancy progressed (Prefumo et al., 2004; Whitley et al., 2007; Melchiorre et al., 2008; Fraser et al., 2012).

### 2.2 Positive selection of dNK cells

Decidual tissue was isolated, washed with HBSS, and dNK cells were isolated using the methods described previously (Fraser et al., 2012). Isolated CD56^+^ dNK cells were cultured at 6 × 10^5^ cells/ml in RPMI 1640 medium (Invitrogen, UK) with 10% (v/v) fetal calf serum (FCS), containing 2.5 μg/ml amphotericin B, 2 mM l-glutamine, 50 μg/ml penicillin, 50 μg/ml streptomycin, 50 ng/ml stem cell factor (SCF) and 5 ng/ml IL-15 (Peprotech, UK) at 37 °C in a 5% CO_2_ humidified incubator. No T cells or macrophages were detected and dNK cell purity was as previously determined (Fraser et al., 2012). Cells were cultured for 24 h after which they were pelleted and lysed for 15 min on ice, in RIPA buffer (50 mM TRIS, pH8, 150 mM NaCl, 1% [v/v] Nonidet P-40, 0.5% [w/v] sodium deoxycholate, 0.1% [w/v] sodium dodecyl sulphate, 1 nM sodium orthovanadate, 1 nM phenylmethyl sulfonyl fluoride, and 10 μg/ml aprotinin). Protein concentration was determined by Bradford assay (BioRad). Conditioned medium was centrifuged for 10 min at 700 × *g* at 4 °C to remove debris. Lysates and culture supernatants were stored at −80 °C. Consistent with our previous studies (Fraser et al., 2012), conditioned medium was pooled from normal-RI dNK and high-RI cultures, matched for the protein concentration in the cell lysates, and used for experiments. There was no significant difference in the gestational ages of the patient samples used in each group.

### 2.3 Culture of the endothelial cell line SGHEC-7

SV40 transfected human umbilical vein endothelial cells (SGHEC-7) (Fickling et al., 1992) were cultured in a 1:1 ratio of Medium 199 supplemented with Earle's modified salts (M199) and RPMI 1640 medium with 10% (v/v) FCS, containing 2 mM l-glutamine, 2.5 μg/ml endothelial cell growth supplement (ECGS), 0.09 mg/ml heparin and 16 mg/ml gentamycin; SGHEC-7 medium.

### 2.4 Detection of ICAM-1 expression by On-cell western assay

4 × 10^4^ SGHEC-7 cells/well were added to a 96-well plate and incubated at 37 °C in an atmosphere of 5% CO_2_ overnight. The SGHEC-7 culture medium was then removed and replaced by 100 μl SGHEC-7 medium (negative control), pooled normal-RI dNK conditioned medium or high-RI dNK conditioned medium (*n* = 14–15) and incubated at 37 °C in an atmosphere of 5% CO_2_ for 24 h. The median gestational age was 11.1 weeks (range 9.3–14.0) for normal-RI and 10.4 weeks (range 9.0–14.3) for high-RI cells used to generate pooled culture supernatants, *p* = 0.2, *t*-test. Cells were then washed, fixed with 4% (w/v) paraformaldehyde in PBS, and blocked overnight with Odyssey® buffer (LI-COR Biosciences, Cambridge, UK). Fixed cells were incubated with 8 μg/ml mouse anti-human ICAM-1 or its control IgG_1_ (R&D Systems, Abingdon, UK), 100 μl/well, for 2.5 h at room temperature. Cells were then washed with PBS and the subsequent protocol using a goat anti-mouse IRDye® 800CW antibody (LI-COR Biosciences) and 1 mM TO-PRO® iodide solution in DMSO (LI-COR Biosciences), was carried out according to the manufacturer's instructions before reading using the Odyssey® infrared-labelled optical imaging system. In each experiment data were normalised such that the ICAM-1 expression with the negative control treatment was given a value of 1.

### 2.5 Enzyme-linked immunosorbent assay for the detection of TNFα

SGHEC-7 cells (1 × 10^6^) were incubated with pooled (*n* = 9) normal-RI or high-RI dNK conditioned medium for 24 h. The median gestational age was 11.6 weeks (range 9.4–13.9) for normal-RI and 10.7 weeks (range 9.4–13.7) for high-RI cells used to generate pools of conditioned medium, *p* = 0.3, *t*-test. Cells were washed three times with PBS and lysed in RIPA buffer. The assay was carried out according to the manufacturer's instructions, using a commercially available enzyme-linked immunosorbent assay (ELISA) kit (TNFα Duoset: R&D Systems, UK and TNFα ELISA development kit: Peprotech, UK).

### 2.6 Endothelial cell network model to assess vessel stability

Wells of Angiogenesis ibiTreat chamber slides (Thistle Scientific, UK) were coated with 10 μl growth factor reduced Matrigel (BD Biosciences, Oxford, UK), and incubated at 37 °C in an atmosphere of 5% CO_2_ for 30 min. 2 × 10^4^ SGHEC-7 cells/well were added (50 μl per well), and allowed to form tube-like structures over 6 h. The SGHEC-7 culture medium was then removed and replaced by 50 μl of pooled normal-RI or high-RI dNK cell conditioned medium (n = 18–19). The median gestational age was 11.3 weeks (range 9.3–13.9) for normal-RI and 10.8 weeks (range 9.1–13.7) for high-RI cells used to generate pools of conditioned medium, *p* = 0.4, *t*-test. To investigate whether dNK-derived factors were inducing EC apoptosis, 50 μM zVAD-fmk, a broad spectrum caspase inhibitor (Calbiochem, UK), was added with normal-RI dNK-conditioned medium. To investigate the effect of TNF-α 20–50 ng/ml recombinant human TNF-α (Peprotech, UK) was added to the preformed EC structures. Images were captured at 0, 2, 5, 10 and 18 h, using an Olympus 1 × 70 inverted microscope (Olympus, Tokyo, Japan) with a Hamamatsu C4742-95 digital camera (Hamamatsu Protonics, UK). At each time point, the length of the EC tube-like structures in duplicate positions of the well were analysed using Image Pro plus software (Media Cybernetics, MD).

### 2.7 Statistical analysis

ANOVA with Bonferroni multiple post-comparison tests, paired or unpaired *t*-tests or Wilcoxon matched pairs signed rank tests were applied as stated (GraphPad Software v5, CA, USA). A *p*-value of <0.05 was considered to be statistically significant.

## 3 Results

### 3.1 Normal-RI dNK cells induce more EC activation than high-RI dNK cells

**Fig. 1:**
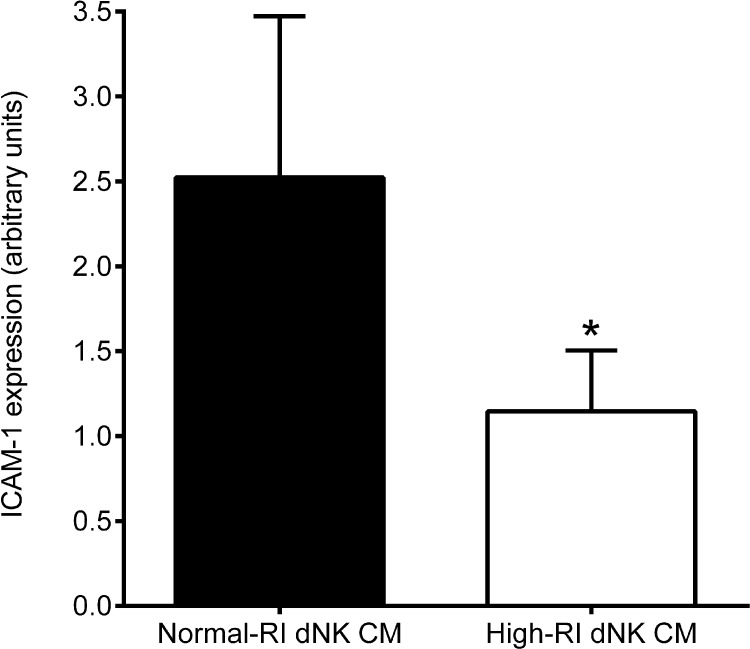
The effect of dNK cell secreted factors on EC ICAM-1 expression. Cell surface ICAM-1 expression (a marker of EC activation) by SGHEC-7 cells after 24-h incubation with pools of normal-RI or high-RI dNK cell-conditioned medium (CM). Results are mean + SEM of experiments carried out with six different pools of each patient group. *p < 0.05; Wilcoxon matched pairs signed rank test.

The HUVEC-derived SGHEC-7 cell line was incubated with pooled normal-RI or high-RI dNK cell conditioned medium for 24 h, followed by measurement of EC ICAM-1 expression by On-cell western. ICAM-1 expression by SGHEC-7 cells was significantly higher (*p* < 0.05) when stimulated with normal-RI dNK cell-conditioned medium compared with those stimulated with high-RI dNK cell-conditioned medium (Fig. 1).

### 3.2 Normal-RI dNK cells induce more EC tube-like structure destabilisation than high-RI dNK cells

**Fig. 2:**
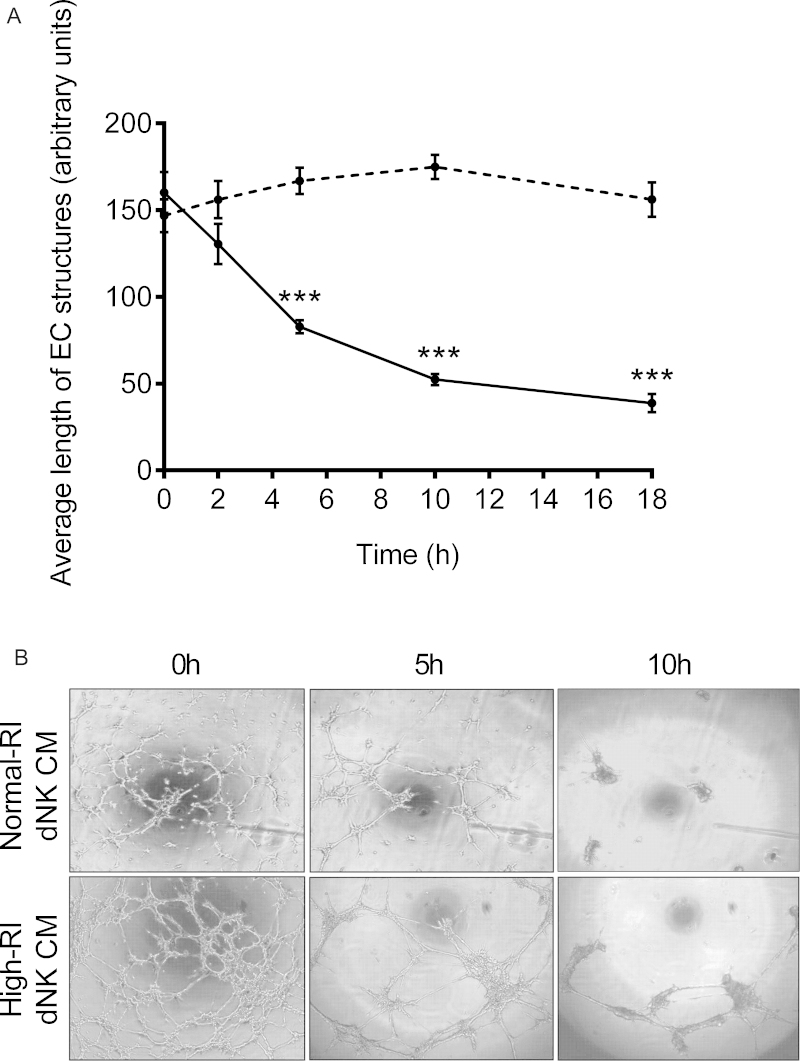
The effect of normal and high-RI dNK secreted factors on EC tube-like structures. SGHEC-7 cells were allowed to form tube-like structures over 6 h, followed by the addition of pooled normal-RI or high-RI dNK-conditioned medium (CM). (A) Average length of EC tube-like structures is determined at 0, 2, 5, 10 and 18 h using Image Pro Plus software. The solid line represents normal-RI dNK CM, the dashed line represents high-RI dNK CM. Results are mean ± SEM of three separate experiments carried out in duplicate. ***p < 0.0001; ANOVA with Bonferroni post-test analysis, normal-RI CM versus high-RI CM at each time point. (B) Images of EC tube-like structures cultured in normal-RI or high-RI dNK CM at 0, 5 and 10 h.

An *in vitro* assay in which ECs form 3D tube-like structures was used to investigate effects on dNK on vessel stability. SGHEC-7 cells were seeded onto growth factor-reduced Matrigel and allowed to form tube-like structures over a period of 6 h. They were then incubated with normal-RI or high-RI dNK-conditioned medium, images were captured at intervals up to 18 h, and the length of the EC tube-like structures determined. Analysis of average EC tube lengths over 18 h indicated that normal-RI dNK-conditioned medium significantly destabilised EC tube-like structures (*p* < 0.0001 at all time points examined after 5 h stimulation, compared with 0 h). This effect was not seen when high-RI dNK-conditioned medium was added to the pre-formed EC tube-like structures (Fig. 2A). Comparison between the effect of the high- and normal-RI conditioned medium showed significant differences at all time points after 5 h. Fig. 2B shows images captured after the addition of normal-RI or high-RI dNK-conditioned medium. In the presence of normal-RI dNK-conditioned medium, the EC structures lost stability over 18 h, resulting in the formation of EC clusters. On treatment with high-RI dNK-conditioned medium, the appearance of the EC structures becomes less intricate and there was a reduction in the number of networks; however, the average length of the network's arms was retained.

**Fig. 3:**
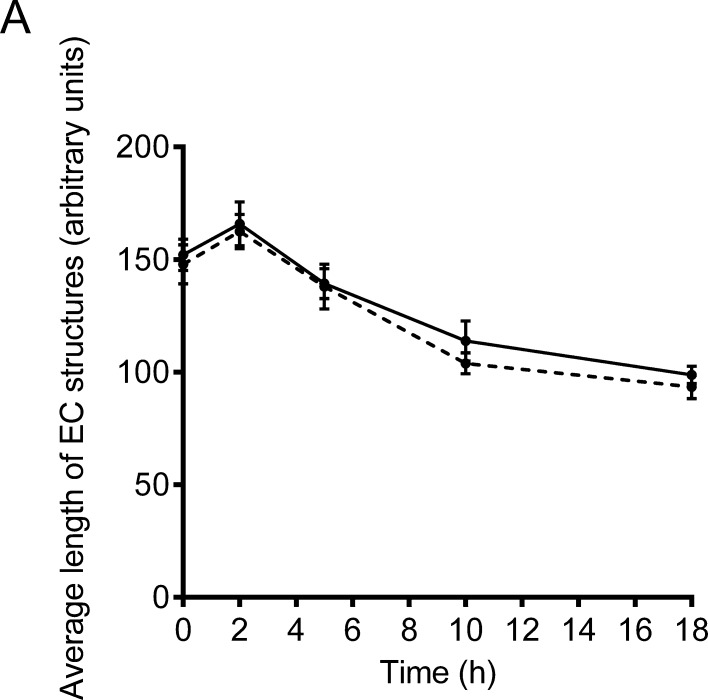
dNK-induced network destabilisation is not caused by apoptosis. SGHEC-7 cells were allowed to form tube-like structures over 6 h, followed by the addition of pooled normal-RI dNK-conditioned medium (CM) ± 50 μM zVAD-fmk. Average length of EC tube-like structures as determined at 0, 2, 5, 10 and 18 h using Image Pro Plus software. Solid line represents normal-RI dNK CM, dashed line represents normal-RI dNK CM with zVAD-fmk. Results are mean ± SEM of three separate experiments carried out in duplicate. Not significant; analysed using an ANOVA with Bonferroni post-test analysis comparing CM with and without zVAD-fmk at each time point.

To determine whether induction of EC apoptosis was contributing to the destabilisation of the EC structures, normal-RI dNK-conditioned medium, containing 50 μM of the broad spectrum caspase inhibitor z-VAD-fmk was added to pre-formed EC tube-like structures and images were captured at intervals up to 18 h. This dose of z-VAD-fmk has been previously shown to inhibit apoptosis of SGHEC-7 cells (Ashton et al., 2005; Fraser et al., 2012). Analysis of the average EC tube lengths showed no significant difference between the lengths of EC structures when treated with normal-RI dNK-conditioned medium compared with the addition of the caspase inhibitor (Fig. 3), suggesting that caspase-dependent apoptosis might not have been involved.

### 3.3 Normal-RI dNK cells induce more EC TNFα production than high-RI dNK cells

**Fig. 4:**
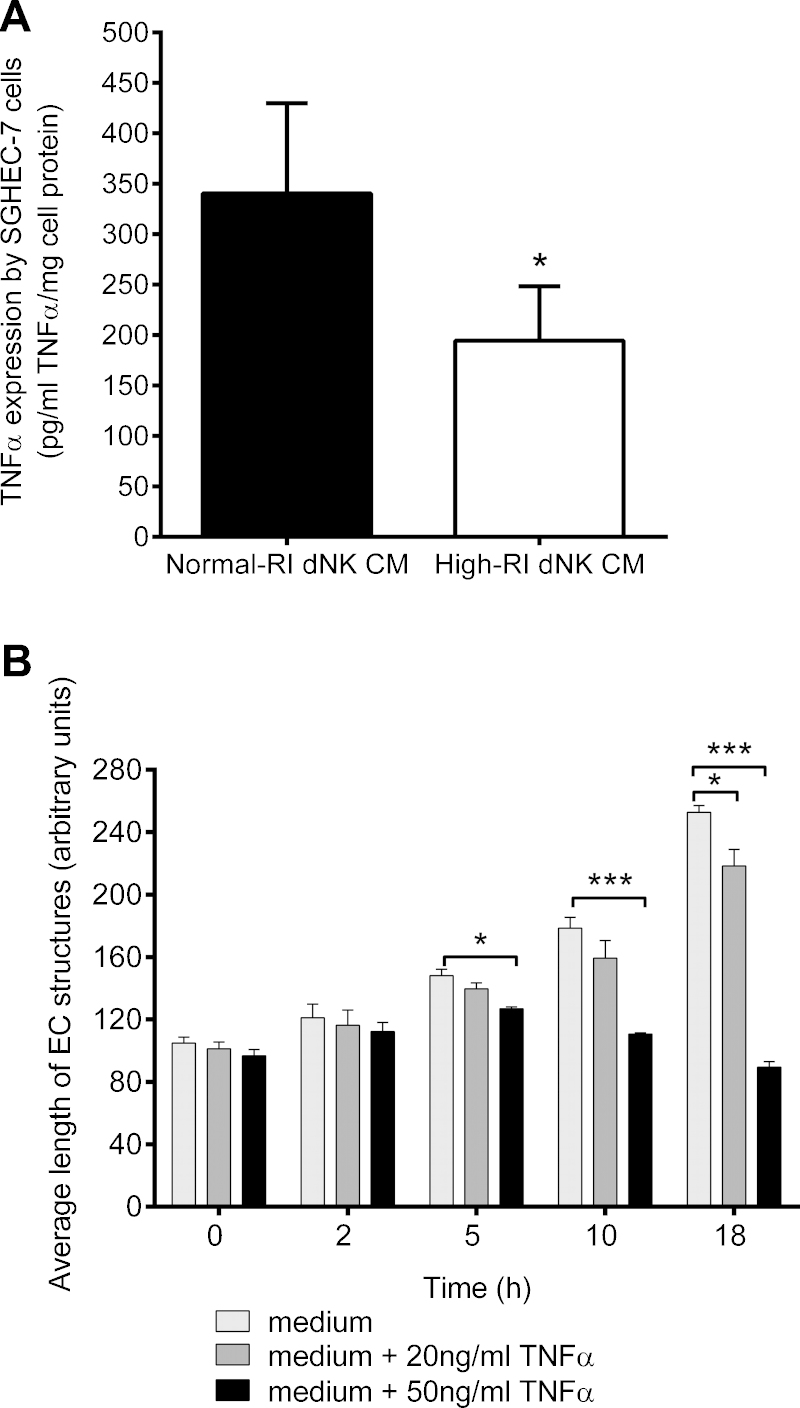
The effect of dNK cell secreted factors on EC TNFα expression. (A) SGHEC-7 cell TNFα expression was determined by ELISA after 24-h incubation with pools of normal-RI or high-RI dNK cell-conditioned medium (CM). Results are mean + SEM of five individual experiments carried out in duplicate. *p < 0.05; paired *t*-test. (B) The effect of TNFα on EC tube-like structures. SGHEC-7 cells were allowed to form tube-like structures over 6 h, followed by the addition of dNK culture medium (not conditioned medium), containing the indicated concentrations of TNFα. Results are mean + SEM of three separate experiments carried out in duplicate. *p < 0.05;**p < 0.001; ***p < 0.0001; ANOVA with Bonferroni post-test analysis compared with medium alone at each time point.

The SGHEC-7 cell line was incubated with pooled normal-RI or high-RI dNK cell-conditioned medium for 24 h, and the cells lysed to examine dNK-secreted factor-induced EC TNFα expression by ELISA. TNFα expression was significantly higher (*p < 0.05) when stimulated with normal-RI dNK cell-conditioned medium than high-RI dNK-conditioned medium (Fig. 4A).

### 3.4 TNFα induces EC tube-like structure destabilisation

The addition of dNK culture medium (not conditioned medium) led to an increase in the average tube length over the time course of the experiment, while the addition of rhTNFα at 50 ng/ml in dNK culture medium significantly reduced average tube lengths after 5 h (*p* < 0.05), 10 h (*p* < 0.001) and 18 h (*p* < 0.0001). Addition of TNFα at 20 ng/ml significantly reduced the average tube length after 18 h (*p* < 0.05; Fig. 4B).

## 4 Discussion

The initial stages of the physiological change in the uterine spiral arteries, such as EC activation and disorganisation, in addition to breaks in the EC layer, take place in the presence of leukocytes, ahead of EVT invasion (Craven et al., 1998; Smith et al., 2009). In this study, we provide evidence that dNK cells from pregnancies with impaired remodelling are less able to activate ECs, determined by EC ICAM-1 expression, which may be one of the contributing mechanisms towards the inefficient spiral artery remodelling seen in these pregnancies.

This study used a culture system in which ECs invade Matrigel to form network structures. Matrigel is rich in laminin and collagen, similar to the extracellular matrix composition of the uterine vessels (Whitley and Cartwright, 2010). The data obtained suggest that dNK-secreted factors might be involved in the destabilisation of endothelial 3D structures, as the tube-like structures were disrupted by dNK cell-conditioned medium, cells became clustered with only short network sprouts remaining, and no connecting networks were retained. Interestingly, factors secreted from dNK cells isolated from high-RI pregnancies (reflecting impaired spiral artery remodelling and a higher risk of developing pre-eclampsia), were less able to destabilise the EC tube-like structures, suggesting that this might contribute to the impaired vascular changes early in the pathology of pre-eclampsia.

Several investigations have demonstrated the involvement of both apoptotic and non-apoptotic mechanisms in the vascular cell changes during spiral artery remodelling (Ashton et al., 2005; Harris et al., 2006; Keogh et al., 2007; Smith et al., 2009; Fraser et al., 2012). We have previously shown that dNK cells, in co-culture with either VSMCs or ECs, induced caspase-dependent apoptotic changes in vascular cells (Fraser et al., 2012). EVT-dependent remodelling of spiral artery ECs, has similarly been suggested to involve apoptotic mechanisms (Ashton et al., 2005; Chen et al., 2005; James et al., 2011). However, the replacement of decidual ECs by EVT may also involve the disruption of inter-endothelial cell junctions, which can cause destabilisation of the EC vessel lining (Bulla et al., 2005). In the current study, a caspase inhibitor had no effect on tube length, suggesting that dNK destabilisation of EC structures does not take place via an apoptotic mechanism. It is possible that secreted factors from dNK might be responsible for the disruption of EC integrity, an important initial event during the spiral artery remodelling process, with apoptotic mechanisms requiring cellular contact with dNK cells or involvement of EVT later on.

Endothelial integrity can be affected by pro-inflammatory cytokines such as TNFα, which can induce endothelial barrier dysfunction, leading to leaky vessels (Menon et al., 2006). The addition of TNFα destabilised vessel structures in our study. We have previously shown that although normal-RI dNK cells express more cell-associated TNFα compared with high-RI dNK cells, the levels of TNFα secreted by cells from the two patient populations do not differ (Fraser et al., 2012). This suggests that it is unlikely to be differences in the levels of TNFα in the conditioned medium that is responsible for the different effects. However, TNFα may also play an autocrine signalling role in the endothelium, as other signals can induce TNFα production by ECs themselves (Neuhaus et al., 2000). We have shown that factors secreted by dNK are able to induce TNFα expression by ECs and that TNFα expression was significantly higher when ECs were stimulated with normal-RI compared with high-RI dNK cell-conditioned medium. As dNK cells isolated from high-RI pregnancies were less able to induce EC destabilisation, we suggest that this might be due to decreased induction of TNFα expression in ECs. Our study has investigated the effect of dNK cells on ECs in the absence of VSMCs, whereas in the vessel environment, the complex cellular interactions in the vessel wall will influence the extent of remodelling that takes place. In this context, it is interesting to consider that TNFα derived from ECs may additionally signal to VSMCs, as TNFα may induce VSMC apoptosis (Geng et al., 1996), and hence further contribute to the remodelling process.

There are several factors in the dNK secretome that differ between the normal-RI and high-RI dNK populations, for example IL-1β (Fraser et al., 2012), and may be responsible for inducing different levels of TNFα expression in ECs (Neuhaus et al., 2000). In addition, several studies have demonstrated that other cytokines, including IL-6, IL-8 and IFN-γ, may influence endothelial integrity, resulting in endothelial barrier dysfunction and vascular permeability (Romer et al., 1995; Sprague and Khalil, 2009). The production of these factors by dNK cells has been previously observed (Hanna et al., 2006; Lash et al., 2006; Fraser et al., 2012). IFN-γ may be of particular interest, as murine studies have demonstrated its importance in dNK-induced remodelling (Ashkar et al., 2000). Further human functional studies will clarify the role of dNK in regulating EC structure through other factors and their involvement in the transformation of human spiral arteries.

In this study, we have established a functional role for dNK cells in the disruption of 3D endothelial structures, we have demonstrated a potential mechanism by which this is occurring, and we have suggested how impairment of this process might be contributing to the reduced vessel remodelling in pregnancies with a high uterine artery resistance index. Although the high-RI group is likely to have aberrant remodelling at this stage, there will be many other factors, such as additional maternal or fetal compensatory mechanisms, that will interact to determine whether the clinical symptoms of pre-eclampsia develop. Our findings may aid further understanding of the cellular interactions between dNK cells and cells of the spiral arteries, and the contribution of these interactions to the pathology of pre-eclampsia, in addition to other pregnancy disorders where remodelling is impaired.

## Conflict of interest

None declared.
